# Ectopic lymphoid neogenesis is strongly associated with activation of the IL-23 pathway in rheumatoid synovitis

**DOI:** 10.1186/s13075-015-0688-0

**Published:** 2015-07-09

**Authors:** Juan D. Cañete, Raquel Celis, Nataliya Yeremenko, Raimon Sanmartí, Leonie van Duivenvoorde, Julio Ramírez, Iris Blijdorp, Carmen M. García-Herrero, José L. Pablos, Dominique L. Baeten

**Affiliations:** Arthritis Unit, Rheumatology Department, Hospital Clinic of Barcelona and IDIBAPS, c/ Villarroel, 170, 08036 Barcelona, Spain; Amsterdam Rheumatology and Immunology Center/Department of Clinical Immunology and Rheumatology, Academic Medical Center/University of Amsterdam, Meibergdreef 9, 1105 AZ Amsterdam, The Netherlands; Rheumatology Department, Instituto de Investigación Hospital 12 de Octubre (I + 12), Avda de Córdoba, s/n, 28041 Madrid, Spain

## Abstract

**Introduction:**

The functional relevance of synovial ectopic lymphoid neogenesis (ELN) in rheumatoid arthritis (RA) remains unknown. As ELN correlates with the degree of tissue inflammation, we investigated whether ELN was associated with specific cytokine profiles.

**Methods:**

Synovial ELN was determined by immunohistology and long CD21 isoform (CD21L) expression. Cytokine expression was determined by multiplex enzyme-linked immunosorbent assay (ELISA) and quantitative polymerase chain reaction (PCR) as well as immunohistology in synovial fluid (SF) (n = 44) and tissue (ST) (n = 108), respectively. Production of ELN-associated chemokines by fibroblast-like synoviocytes (FLS) was studied in vitro.

**Results:**

Screening analysis of SF by multiplex ELISA showed higher protein levels of interleukin (IL)-23 (*p* = 0.018) and IL-17F (*p* = 0.028) in ELN+ versus ELN- samples. Other cytokines, including IL-17A, IL-6, and tumor necrosis factor (TNF)-α, were not different. The association between IL-23 and ELN was not biased by disease activity or other clinical features and was confirmed by higher IL-23 mRNA expression in ELN+ versus ELN- ST samples (*p* = 0.030), a correlation between IL-23 and CD21L expression in the same samples (r = 0.70 *p* < 0.0001), and a similar correlation in two independent ST sample sets (r = 0.778 *p* < 0.0001 and r = 0.817 *p* = 0.011). IL-23 p19 staining was neither restricted nor enhanced in close proximity of ectopic lymphoid follicles, and neither IL-23 nor IL-17A stimulation induced expression of the ELN-associated CC chemokine ligand, CCL21 and CXC chemokine ligand CXCL13, by FLS. Downstream of IL-23, CD21L expression was significantly associated with IL-17F, IL-21, and IL-22, but not IL-17A in two independent ST sample sets.

**Conclusions:**

Synovial ELN in RA is strongly associated with activation of the IL-23 pathway but not with IL-17A.

**Electronic supplementary material:**

The online version of this article (doi:10.1186/s13075-015-0688-0) contains supplementary material, which is available to authorized users.

## Introduction

Ectopic lymphoid neogenesis (ELN) is a histological feature describing the organized aggregation in nonlymphoid tissues of T and B lymphocytes and, in a proportion of cases, follicular dendritic cells (FDC) around newly developed vessels called high endothelial venules (HEV), which are similar to those found in lymphoid organs [1, 2]. ELN has been described in multiple different organs and tissues during inflammation, including synovial tissue (ST) in rheumatoid arthritis (RA) as well as other forms of inflammatory arthritis [3–5]. ELN is associated to in situ expression of cytokines and chemokines such as lymphotoxin (LT)β, CXC chemokine ligand (CXCL)13 and CC chemokine ligand (CCL)21 [6, 7] and the expression of these factors is sufficient to induce ELN in transgenic models [8–10]. The specific microarchitecture and cellular composition of ELN suggests a role in immune responses and potentially autoimmunity [5, 11]. Although experimental evidence indicates that ELN+ ST can produce autoantibodies [12], a series of large translational studies failed to demonstrate a link between synovial ELN and systemic or local RA-specific antibodies [5, 13, 14]. Moreover, the presence of synovial ELN was not associated with a clear clinical or pathological RA phenotype as associations with disease activity, disease severity and response to anti-tumor necrosis factor (TNF)-α treatment were weak and inconsistent [5, 13, 14]. Accordingly, the pathogenic and clinical significance of synovial ELN still remains unclear.

As we previously observed a clear association between ELN and the histological degree of inflammatory infiltration in ST [5, 13], we postulated that there might be an autoantibody-independent link between ELN and synovial inflammation. In line with emerging evidence that B cells are potent cytokine producers and antigen-presenting cells [15, 16], it was reported that T cell activation in rheumatoid synovitis is B cell-dependent [17]. We therefore hypothesized that the specific T/B cell organization observed in synovial ELN may be associated with a specific T cell cytokine profile. To test this hypothesis we investigated the local expression of T helper (Th)1, Th2, Th17, and proinflammatory cytokines in RA patients with and without synovial ELN.

## Methods

### Patients and synovial tissues

Synovial biopsy specimens were obtained by needle arthroscopy in 108 patients fulfilling the American College of Rheumatology (ACR) criteria for RA [18] who had clinically active arthritis of at least one knee joint. None of the patients had been treated with biological therapy at time of inclusion. Arthroscopy was performed under diagnostic and/or therapeutic (lavage) indication with a 2.7 mm arthroscope (Storz, Tullingen, Germany). Eight samples were obtained from the suprapatellar pouch and the medial and lateral gutter in each patient [14]. Four samples were fixed in 4 % formaldehyde and embedded in paraffin wax for immunohistochemistry and the remaining four collected on RLT lysis buffer (Qiagen, Crawley, UK) for RNA extraction.

A first cohort, used for exploratory analysis of correlations between ELN and cytokine expression, consisted of 63 RA patients. Demographics and clinical data are summarized in Table 1. In a subgroup of these patients (n = 44), synovial fluid (SF) collected at the time of the arthroscopy was centrifuged and frozen at −80 °C. Additional synovial biopsies were obtained from an independent confirmation cohort of 36 RA patients fulfilling the ACR criteria (72 % female, mean age 65 years) with a mean 28-joint disease activity score (DAS28) of 5.6 and a mean C-reactive protein (CRP) of 1.53 mg/dL. Finally, synovial biopsies for analysis of T cell transcription factors were obtained from a third independent cohort consisting of nine RA patients fulfilling the ACR criteria (89 % female, mean age 57 years) with a mean DAS28 of 7.3 and a mean CRP of 6.69 mg/dL). All patients gave written informed consent. The present study was approved by the institutional ethics committee of both participating centers (Clinical Research Ethics Committee of the Hospital Clinic of Barcelona, Barcelona, Spain, and Medical Ethics Committee of the Academic Medical Center/University of Amsterdam, Amsterdam, the Netherlands).

**Table 1:** Demographic and clinical data of RA patients from cohort 1 stratified by presence or absence of ectopic lymphoid neogenesis (ELN)

	Total (*n* = 63)	ELN+	ELN–	*p* value
		*n* = 30 (47.6 %)	*n* = 33 (52.4 %)	
Female, n (%)	41 (65)	21 (70)	20 (61)	NS
Age (years)	59 (49; 69)	56 (50; 64)	62 (49; 70)	NS
Disease duration (years)	12.0 (4.8; 17.8)	9.1 (3.9; 17.8)	13.1 (10.5; 17.8)	NS
Tender joint count	3 (1; 10)	4 (2; 10)	2 (1; 10)	NS
Swollen joint count	5 (2; 9)	6 (2; 10)	3 (1; 8)	NS
C-reactive protein (mg/dL)	2.4 (1.1; 4.2)	2.7 (1.4; 5.1)	2.1 (0.7; 3.8)	NS
ESR (mm/h)	33 (19; 67)	35 (19; 67)	31 (18; 53)	NS
DAS28	4.4 (3.3; 5.5)	5.1 (3.9; 5.7)	4.0 (3.0; 4.9)	0.039
RF (IU)	128 (24; 257)	138 (61; 256)	74 (17; 325)	NS
ACPA (IU)	254 (0; 782)	257 (0; 701)	251 (0; 822)	NS
Number of DMARDs taken before biopsy	2 (1; 3)	2 (1; 3)	2 (1; 4)	NS
Number (%) of patients taking oral steroids^a^	46 (73)	23 (76)	23 (70)	NS

Data are expressed as median (IQR), or frequency (%)

*NS* nonsignificant, *ESR* erythrocyte sedimentation rate, *DAS28* 28-item disease activity score, *RF* rheumatoid factor, *IU* international units, *ACPA* anti-citrullinated peptide/protein antibodies, *DMARDs*, disease-modifying antirheumatic drugs

^a^All of them ≤ 5 mg prednisone

### Immunohistochemistry

Sequential sections of RA ST were analyzed for the presence of lymphoid aggregates and the expression of the following markers by peroxidase immunohistochemical analysis: T cells were labeled with rabbit anti-human CD3 polyclonal antibody (A0452, Dako, Cambridge, UK), B cells with mouse anti-human CD20 antibody (clone L26, Dako), HEV with rat anti-human peripheral lymph node addressin (PNAd) antibody (clone MECA-79, PharMingen, Oxford, UK) and macrophages with MoA anti-CD68 (clone KP-1, Dako). Antigen retrieval, which was required for most antibodies, was performed by microwave heating in 1 mM ethylenediaminetetraacetic acid (EDTA) for 15 min. Primary antibodies were detected with appropriate secondary biotinylated antibodies using a biotin peroxidase-based method (ABC, Vector Laboratories, Burlingame, CA, USA) and diaminobenzidine as the chromogen. Parallel sections were incubated with irrelevant isotype- and concentration-matched monoclonal antibodies as negative control. Sections were finally counterstained in Gill’s hematoxylin.

### Analysis of lymphoid aggregates

The highest grade of lymphoid aggregation within each sample was determined according to a previously described scoring method [6] based on the number of radial cell counts: grade 1 = 2–5 radial cell counts, grade 2 = 6–10 radial cell counts, and grade 3= > 10 radial cell counts. T/B cell segregation and PNAd-positive HEV within lymphoid aggregates was analyzed. ELN was histologically defined as the presence of follicular aggregates grade ≥ 2 with T/B cell segregation and HEV. To define ELN at the molecular level, we performed quantitative real-time polymerase chain reaction (qRT-PCR) for CD21 long isoform (CD21L). This isoform of CD21 confers unique functions of FDCs in germinal center development [19] and is selectively expressed in germinal center-containing synovial tissues [1].

### Quantification of cytokines in synovial fluid

SF cytokines were analyzed using Quantibody® Human TH17 Array 1 (granulocyte macrophage colony-stimulating factor (GM-CSF), interleukin (IL)1-β, IL-2, IL-4, IL-5, IL-6, IL-10, IL-12p70, IL-13, IL-17A, IL-17F, IL-21, IL-22, IL-23, interferon gamma (IFNγ), CCL20, transforming growth factor beta 1 (TGF-β1), TNF-α and TNF-β) (RayBiotech, Norcross, GA, USA) according to the manufacturer’s specifications. Each sample was prepared in quadruplicate. An Axon scanner 4000B with GenePix software (Molecular Devices, Sunnyvale, CA, USA) was used to collect fluorescence intensities. Detection limits for cytokines are displayed on the manufacturer’s website [20].

### Real-time quantitative PCR

Total RNA was extracted from ST samples according to the recommendations of the RNeasy FFPE Kit (Qiagen, Crawley, UK). The quality of the RNA was assessed by nanodrop (NanoVue Plus, General Electric, Frieburg, Germany) and 1–2 micrograms of RNA was transcribed into complementary deoxyribonucleic acid (cDNA) using a high-capacity cDNA Archive Kit (Applied Biosystems, Warrington, UK). TaqMan gene expression assays for human glyceraldehyde-3-phosphate dehydrogenase (GAPDH) (4310884E), CD21L (Hs00153398), TNF-α (Hs00174128_m1); IL-1β (Hs01555410_m1), IFN-γ (Hs00989291_m1), IL-2 (Hs00174114_m1), IL-6 (Hs00174131_m1), IL-17A (Hs00936345_m1), IL-17F (Hs00369400_m1), IL-21 (Hs00222327_m1), IL-22 (Hs01574154_m1), IL-23 (Hs00372324_m1), RAR-related orphan receptor C (RorC) (Hs01076122_m1), T-box 21 (TBX21) (Hs00203436_m1), GATA binding protein 3 (Gata3) (Hs00231122_m1), aryl hydrocarbon receptor (Ahr) (Hs00169233_m1), forkhead box P3 (FoxP3) (Hs01085834_m1) and B-cell CLL/lymphoma 6 (Bcl6) (Hs00153368_m1) were purchased from Applied Biosystems, and gene expression was measured in duplex reactions. The relative expression (represented in arbitrary units, a. u.) was calculated with the “2^(−ddCt) method”, where dCt = Ct_gene_-Ct_housekeeping gene_, ddCt = dCt_sample_-dCt_calibrator_. In our analysis, we used GAPDH as a housekeeping gene and one of the samples as an internal calibrator [21]. The results were calculated using the StepOne Software v 2.1 (Applied Biosystems).

### Immunofluorescence

Frozen synovial biopsy tissue sections were used for double staining of IL-23-positive cells. The staining was performed with monoclonal mouse anti-human IL-23 p19 (clone eBio473P19, eBioscience, San Diego, CA, USA) in combination with biotinylated monoclonal antibodies against macrophages (CD68, clone Y1/82A, Biolegend, San Diego, CA, USA) and stromal cells (vimentin, (D21H3) XP™ rabbit monoclonal antibody, Cell Signaling Technology, Danvers, MA, USA). Incubation with the primary antibodies was carried out overnight at 4 °C, followed by incubation with a secondary Alexa Fluor 488-conjugated goat anti-mouse antibody and streptavidin-Alexa Fluor 594 (in case of staining with CD68) and secondary Alexa Fluor 594-conjugated goat anti-rabbit antibody (in case of staining with vimentin) was used. Slides were mounted with Vectashield containing 4′,6-diamidino-2-phenylindole (DAPI) (Vector Laboratories) and analyzed on a fluorescent imaging microscope (Leica DMRA, Wetzlar, Germany) coupled to a CCD camera and Image-Pro Plus software (Media Cybernetics, Dutch Vision Components, Breda, the Netherlands).

### Fibroblast-like synoviocytes cultures

Fibroblast-like-synoviocytes (FLS) cells were grown in Dulbecco’s modified Eagle’s medium (Lonza, Basel, Switzerland) containing 4.5 g/l glucose and supplemented with 10 % fetal bovine serum (FBS), 2 mM glutamine, penicillin (100 u/ml) and streptomycin (100 mg/ml). The cells were maintained in a humidified atmosphere containing 5 % CO_2_ at 37 °C.

Triplicate cultures from three different FLS lines from RA patients (1.2 × 10^4^ cells/cm^2^) were seeded in 6-well culture dishes at passage 5–6 and allowed to adhere overnight. Thereafter, the cells were cultured in the absence or presence of recombinant human IL-17A (R&D Systems, Minneapolis, MN, USA) or IL-23 (Miltenyi Biotech, Bergisch Gladbach, Germany) (10 ng/ml) for 24 hours in FLS culture medium. Nonstimulated cells served as control.

Relative expression levels of CXCL13 and CCL21 messenger ribonucleic acid (mRNA) were determined by qRT-PCR. Induction of RANKL or MMP-1 mRNA was used as positive control for the response to IL-23 and IL-17, respectively [22, 23]. Total RNA from FLS was isolated using TRIReagent (Sigma-Aldrich, St Louis, MO, USA) according to the manufacturer’s instructions. The RNA concentration and quality were determined with a Nanodrop ND-1000 spectrophotometer (Thermo Fisher Scientific, Waltham, MA, USA). Subsequently, 1 μg RNA was reverse transcribed using High-Capacity cDNA Reverse Transcription Kit (Applied Biosystems). qRT-PCR were performed by Applied Biosystems 7500 Fast Real-Time PCR System using Power Syber Green PCR Master Mix. Relative gene expression was determined as described previously [24].

### Statistical analysis

Numerical variables were described as median and interquartile range (IQR) and categorical variables as frequencies and percentages. The Wilcoxon rank-sum test or Mann–Whitney test were used to compare the distribution of numerical variables between groups. Fisher’s exact test was used to compare categorical variables. Correlation between numerical variables was expressed using Spearman’s correlation coefficient. Correlation between two categorical variables or between one numerical and one categorical variable was assessed using Fisher’s exact test and the Wilcoxon rank-sum test. As single cytokines were correlated with ELN but not with each other, Bonferroni correction for multiple dependent correlations was not applied; confirmation of the findings in an independent sample set was used to exclude false-positive findings. All analyses were performed using the SPSS v14 (SPSS Inc., Chicago, IL, USA) and STATA v10 (StataCorp, College Station, TX, USA) programs, except for SF cytokines. Cytokine data analysis was performed using SPSS 18.0 (IBM Corp, Armonk, NY, USA).

## Results

### Histological and molecular characterization of synovial ectopic lymphoid neogenesis

**Fig. 1:**
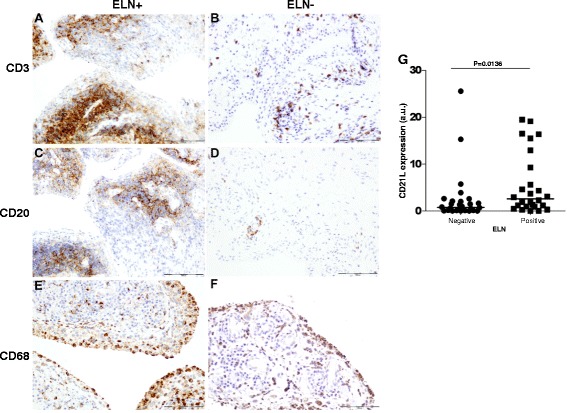
Immunohistological analysis of the cellular infiltrate in synovial membrane of RA patients from cohort 1 stratified according to ectopic lymphoid neogenesis (ELN). **a** and **b** CD3+ lymphocytes. **c** and **d** CD20+ B lymphocytes. **e** and **f** CD68+ macrophages. **g** Relative mRNA expression of CD21L in histologically defined ELN (negative or positive). *CD21L* CD21 long isoform, *mRNA* messenger ribonucleic acid, *RA* rheumatoid arthritis

Thirty out of sixty-three patients (48 %) of the exploratory cohort (Table 1) had synovial ELN defined as the presence of follicular aggregates grade ≥ 2 with T/B cell segregation and HEV. There were no demographic or clinical differences between ELN+ and ELN- patients with exception of a higher DAS28 score in ELN+ patients (median 5.1; range 3.9–5.7) versus ELN- patients (4.0; 3.0–4.9) (*p* = 0.039) (Table 1). However, other measures of disease activity such as C-reactive protein (CRP), erytrocyte sedimentation rate (ESR), swollen joint count (SJC) and tender joint counts (TJC) were not different between both groups. In agreement with previous studies [5, 13, 14], there were also no differences in disease duration and in presence and/or titers of rheumatoid factor (RF) and anti-citrullinated peptide/protein antibody (ACPA) (Table 1). Histological analysis showed a significantly increased infiltration with CD3+ T lymphocytes (*p* < 0.001) (Fig. 1a and b) and CD20+ B lymphocytes (*p* < 0.001) (Fig. 1c and d) in ELN+ versus ELN- synovial tissue samples. Infiltration of the synovial lining layer and the synovial sublining with CD68+ macrophages was similar in both groups (Fig. 1e and f). Analysis of CD21L mRNA as molecular marker for ELN confirmed significantly increased expression levels in ELN+ versus ELN- synovial tissue samples (*p* = 0.014) (Fig. 1g).

### Increased synovial fluid levels of IL-23 and IL-17F in the presence of synovial ectopic lymphoid neogenesis

**Fig. 2:**
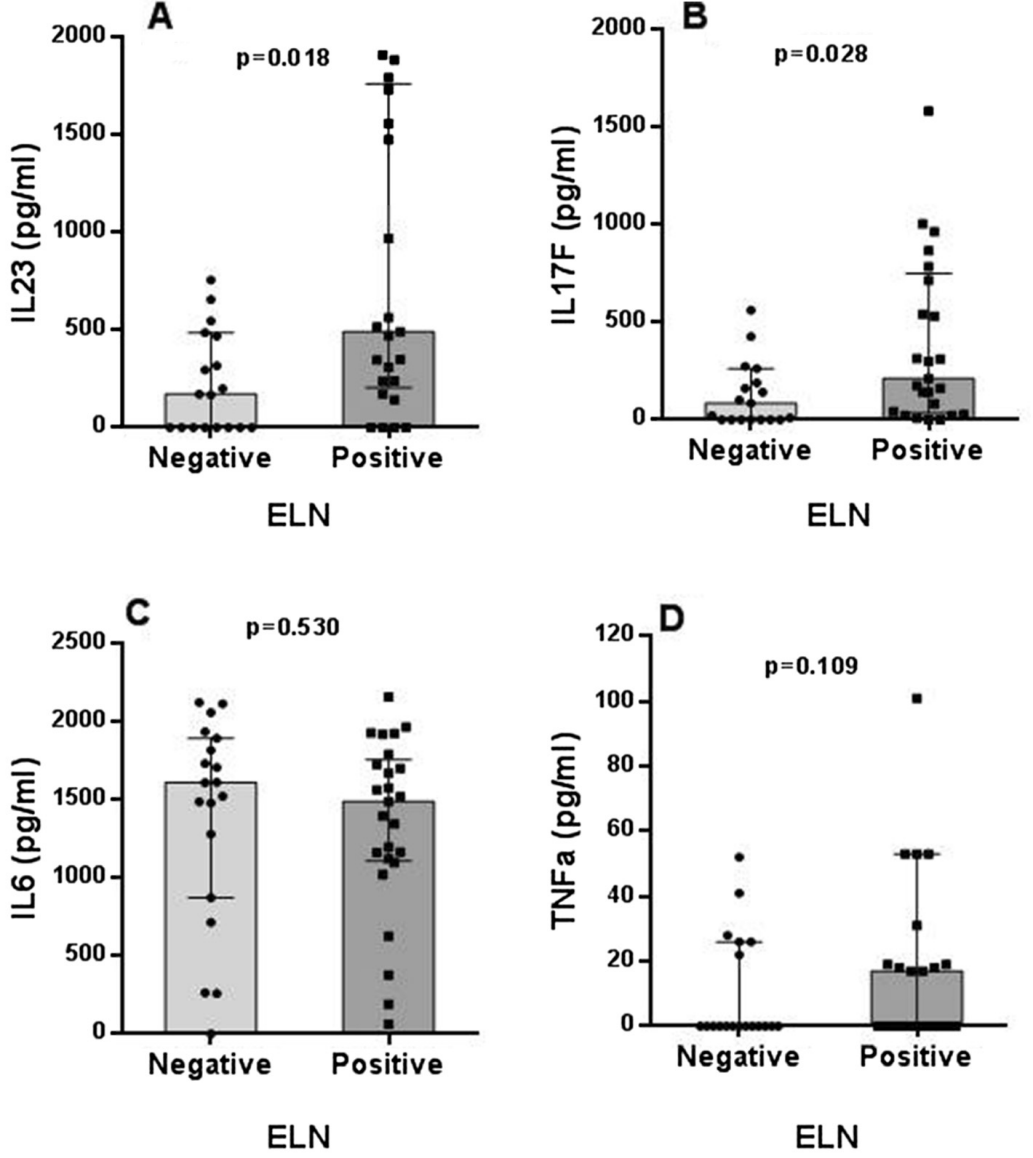
Synovial fluid cytokine levels as determined in RA patients from cohort 1 stratified according to ectopic lymphoid neogenesis (ELN). IL-23 (**a**), IL-17F (**b**), IL-6 (**c**) and TNFa (**d**). Median and interquartile ranges are represented. *RA* rheumatoid arthritis. TNF-a: Tumor necrosis factor alpha-

To assess whether synovial ELN is associated with a specific proinflammatory cytokine profile, we first assessed SF levels of Th1, Th2, Th17 and proinflammatory cytokines in 25 ELN+ versus 19 ELN- samples. The expression of many cytokines including the Th2 cytokines IL-4, IL-5 and IL-13 were low to undetectable in SF independently of the presence of ELN (data not shown). Strikingly, however, SF levels of IL-23 (*p* = 0.018) and IL-17F (*p* = 0.028) were significantly increased in ELN+ versus ELN- samples (Fig. 2a and b). There was also a numerical but not statistically significant increase in IL-22 (*p* = 0.070), TGF-β1 (*p* = 0.060), and IFN-γ (*p* = 0.052) (data not shown). Other proinflammatory cytokines implicated in RA inflammation such as IL-6 and TNF-α (Fig. 2c and d) were not different between both groups, indicating that the increase in IL-23 and IL-17F is not merely reflecting increased local disease activity in ELN+ synovitis.

### Confirmation of the association between IL-23 and synovial ectopic lymphoid neogenesis

**Fig. 3:**
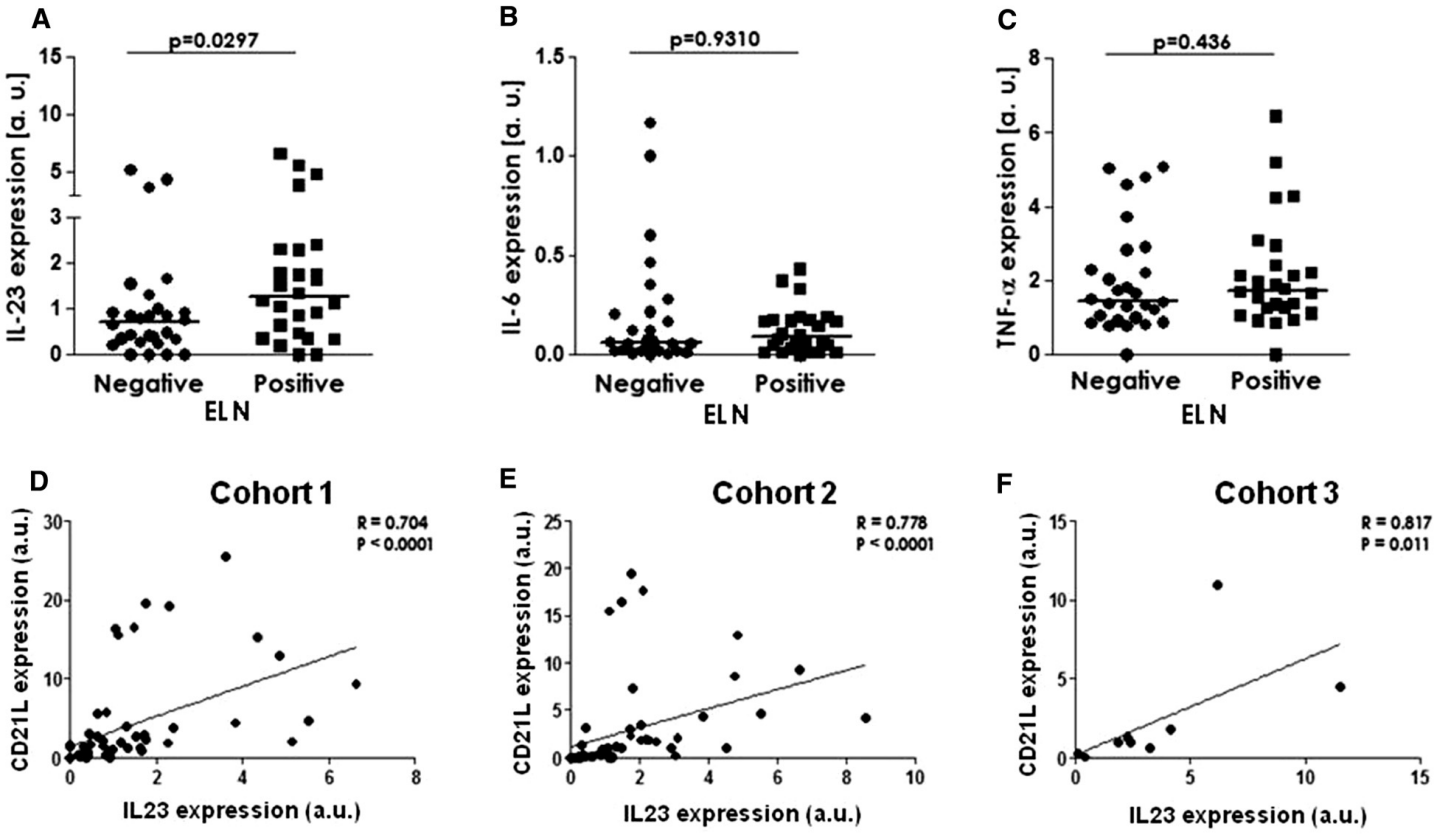
Analysis of synovial tissue mRNA expression of IL-23 (**a**), IL-6 (**b**) and TNF-α (**c**) by quantitative PCR in the cohort 1 of RA. Analysis with ectopic lymphoid neogenesis (ELN) based on the histology. Correlation of mRNA expression of CD21L (molecular marker of ELN) with expression of IL-23 in the three RA cohorts (**d-f**). *CD21L* CD21 long isoform, *IL* interleukin, *mRNA* messenger ribonucleic acid, *PCR* polymerase chain reaction, *RA* rheumatoid arthritis, *TNF-α* tumor necrosis factor alpha

As the SF screening revealed elevated IL-23 levels in ELN+ versus ELN- samples, we next performed a series of experiments to confirm this association. First, quantitative polymerase chain reaction (PCR) on synovial tissue biopsies indicated increased mRNA expression of IL-23 (*p* = 0.030) in ELN+ (n = 30) versus ELN- (n = 33) samples from cohort 1 (Fig. 3a). Again, this association was specific for IL-23 as expression levels of the proinflammatory cytokines IL-6 and TNF-α were not different between both groups (Fig. 3b and c). Second, IL-23 mRNA expression was also significantly correlated with mRNA expression of CD21L as molecular marker of ELN (r = 0.70; *p* < 0.0001) (Fig. 3d), thereby excluding a potential bias by histological misclassification of samples with regard to ELN. Third, this association was not biased by disease activity as there was no significant correlation between ST expression of IL-23 and measures of disease activity such as DAS28 and CRP (data not shown). Finally, we could confirm the correlation between synovial CD21L mRNA and IL-23 mRNA expression in two additional and independent sample sets [r = 0.778, *p* < 0.0001 in the first confirmation cohort of 36 synovial tissue samples (cohort 2) (Fig. 3e) and r = 0.817, *p* = 0.011 in the second confirmation cohort of nine synovial tissue samples (cohort 3) (Fig. 3f)]. Collectively, the different approaches and the use of different independent cohorts confirmed a robust, technically and biologically reproducible correlation between synovial IL-23 and ELN.

### Exploration of the functional relationship between IL-23 and ectopic lymphoid neogenesis

**Fig. 4:**
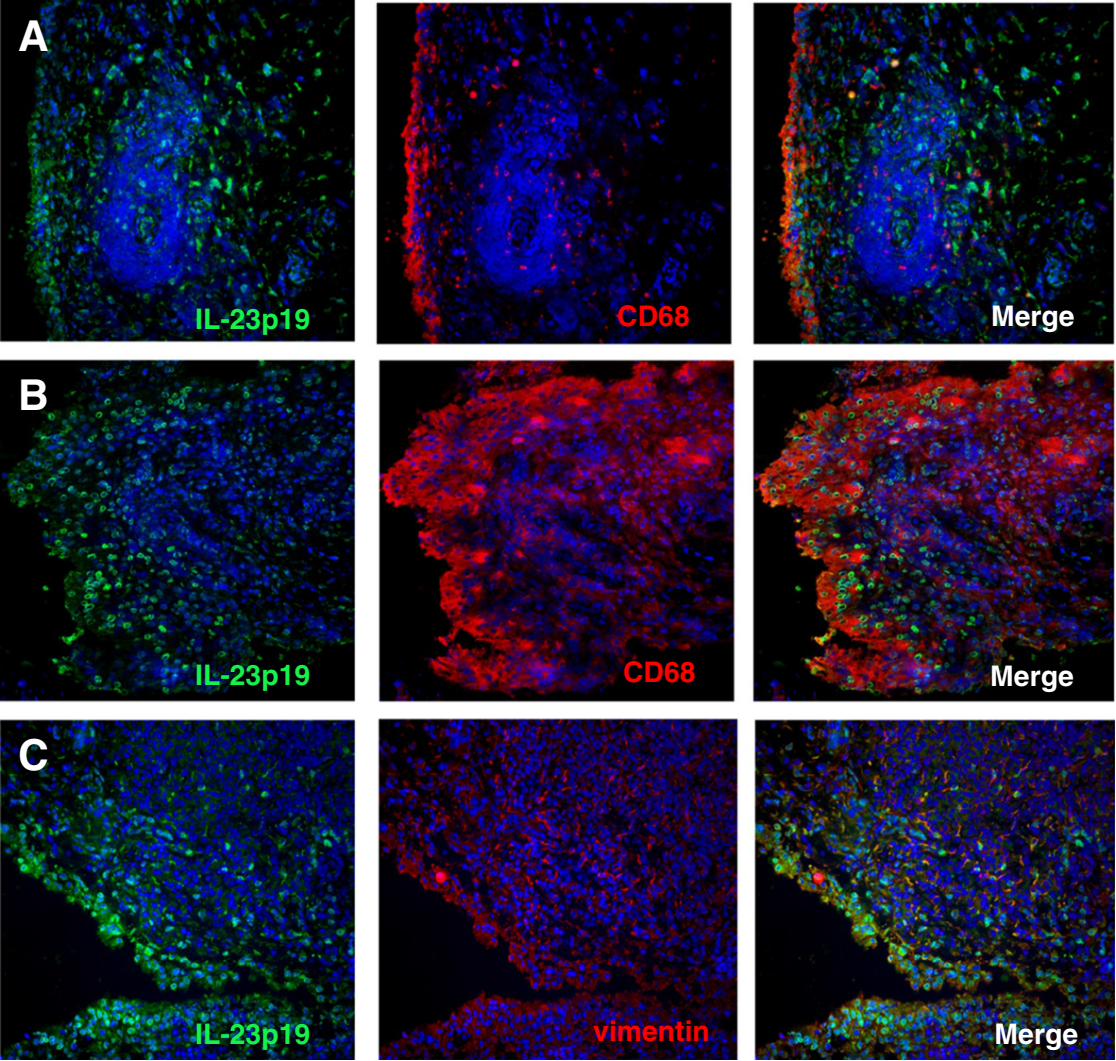
Cellular distribution of IL-23 in synovial membrane of RA patients. Double immunofluorescence analysis in RA synovium was performed using specific antibodies against IL-23p19 (*green*), CD68 or vimentin (*red*). DAPI- (*blue*) stained nuclei. p19 double staining with CD68+ macrophages showing that ELN does not form a microarchitectural niche for IL-23-producing cells (a and b). IL-23 expression by vimentin-positive stromal cells (c). Magnification × 25. *DAPI* 4′,6-diamidino-2-phenylindole, *IL* interleukin, *RA* rheumatoid arthritis. ELN: ectopic lymphoid neogenesis

**Fig. 5:**
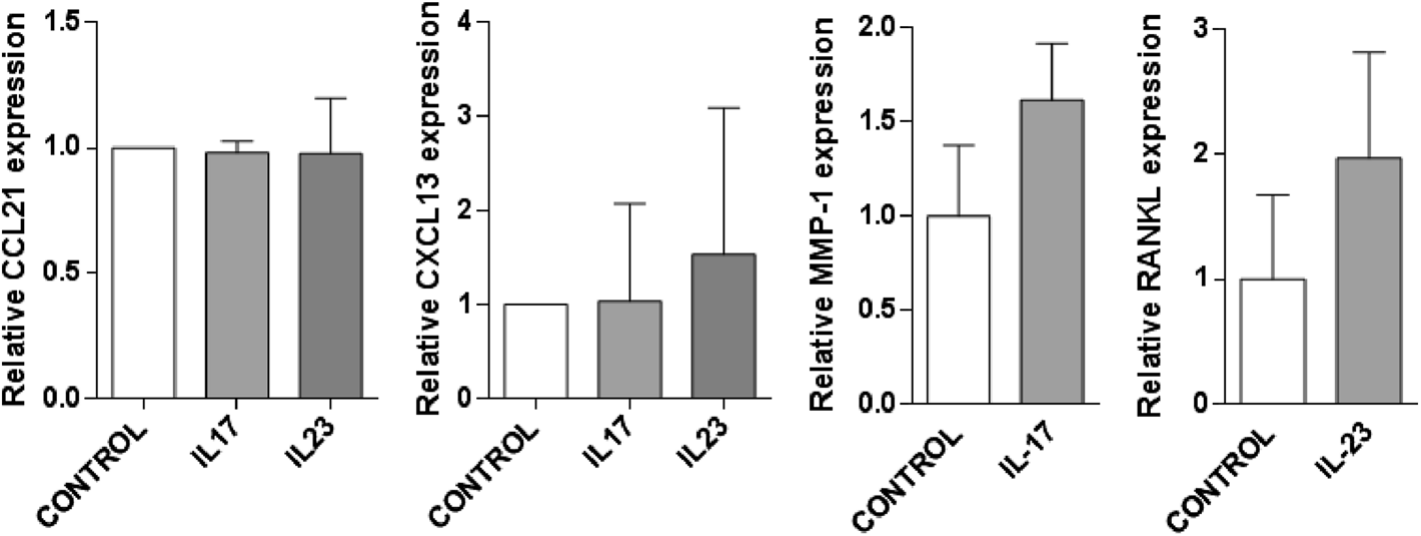
Relative expression levels of CXCL13 and CCL21 were determined by quantitative real-time-PCR (qRT-PCR). Induction of RANKL or MMP-1 mRNA was used as positive control for the response to IL-23 or IL-17, respectively. *CCL* CC chemokine ligand, *CXCL* CXC chemokine ligand, *IL* interleukin, *mRNA* messenger ribonucleic acid

In order to explore if synovial ELN might be IL-23-dependent, we first immunostained IL-23 protein in synovial biopsy samples. As shown in Fig. 4, clear staining for IL-23 was detected throughout the synovial biopsy sections. The p19 staining was neither restricted nor enhanced in close proximity of ectopic lymphoid follicles, indicating that ELN does not form a microarchitectural niche for IL-23-producing cells (Fig. 4a and b). Interestingly, double staining indicated that the IL-23-specific p19 subunit was not only expressed by CD68+ macrophages but also by vimentin-positive stromal cells (Fig. 4c). Control stainings with isotype control confirmed the specificity of the anti-p19 stainings (Figure S1 in Additional file 1) We next investigated if IL-23 or IL-17A stimulation of FLS in vitro leads to increased production of CCL21 and CXCL13, two pivotal chemokines in ELN. CCL21 and CXCL13 mRNA expression was clearly detected under basal conditions in cultured RA FLS, albeit CXCL13 expression was highly variable among different FLS lines. However, neither CCL21 nor CXCL13 mRNA expression were significantly induced by either IL17A or IL23 stimulation (Fig. 5). Thus, these in vitro experiments did not provide clear evidence that ELN is IL-23 dependent, but more thorough in vivo analysis remains warranted.

### Expression of IL-23-dependent cytokines in the presence of ectopic lymphoid neogenesis

**Fig. 6:**
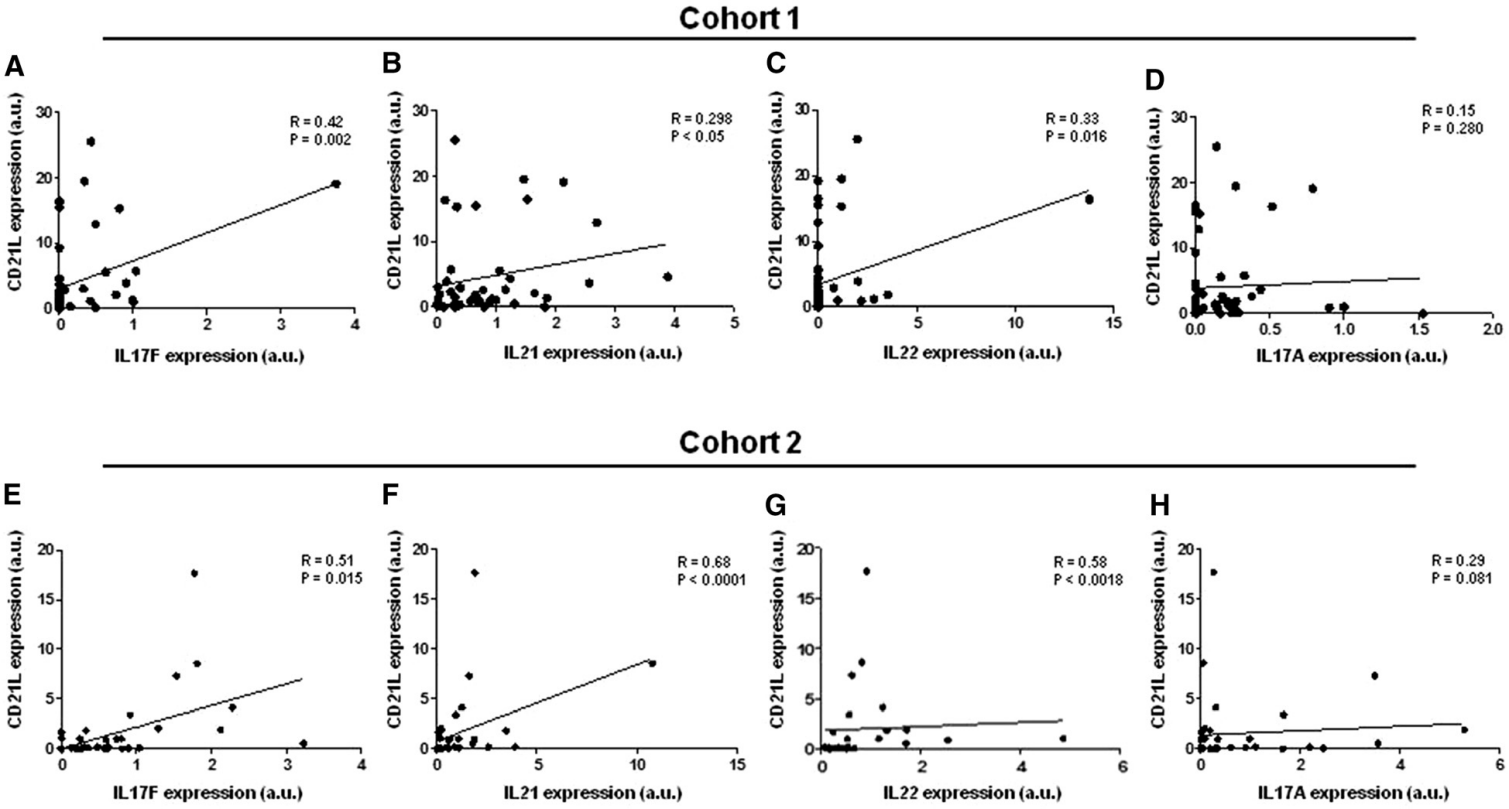
Correlation between mRNA expression of CD21L (molecular marker of ELN) and Th17-related cytokines in the synovial tissue of cohort 1 (**a-d**) and cohort 2 (**e-h**). *CD21L* CD21 long isoform, *ELN* ectopic lymphoid neogenesis, *mRNA* messenger ribonucleic acid, *Th* T helper

**Fig. 7:**
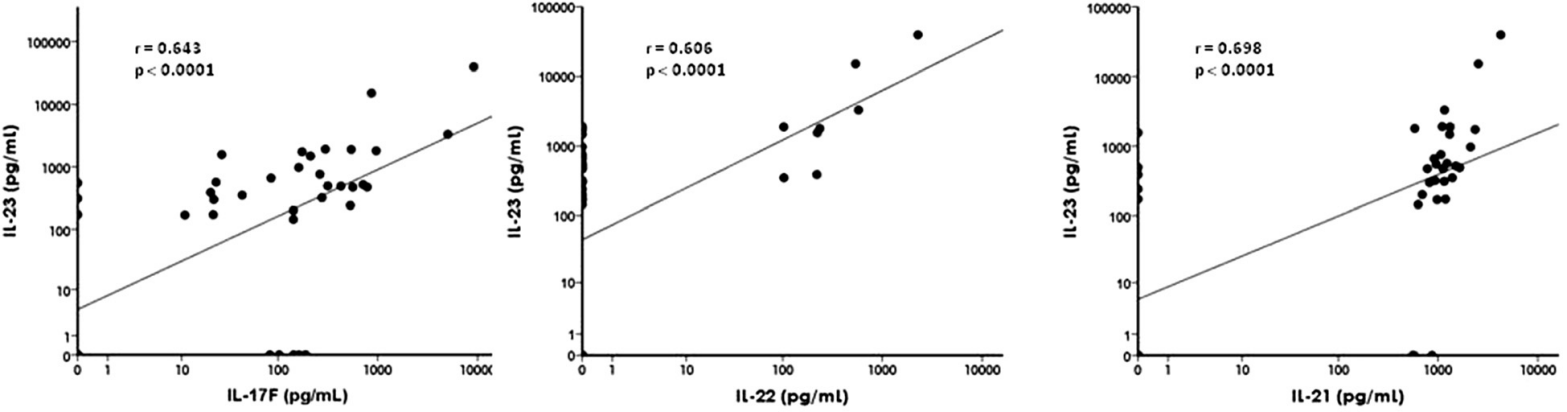
Spearman’s correlation between synovial fluid cytokine levels of IL-23 with IL-17F, IL-22 and IL-21 as determined in RA patients. Logarithmic scale representation. *IL* interleukin, *RA* rheumatoid arthritis

We next investigated if the increased expression of IL-23 in ELN+ synovial tissue was associated with a skewing of T cell cytokines toward a Th17 profile. In agreement with the increased SF IL-17F levels in ELN+ samples, mRNA expression analysis of synovial tissue confirmed that CD21L was significantly correlated with IL-17F (r = 0.42; *p* = 0.002) but not IL-17A (Fig. 6a and d). Moreover, there was a clear correlation with IL-21 (r = 0.3; *p* = 0.049), and IL-22 (r = 0.33; *p* = 0.016) expression (Fig. 6b and c). Investigating the discrepancy between IL-17A and other IL-23-dependent cytokines, we first repeated the same analysis in our validation cohort (cohort 2). Also in this independent sample set, CD21L as molecular marker of ELN showed a significant correlation with IL-17F (r = 0.51; *p* = 0.015), IL-21 (r = 0.68; *p* < 0.0001), IL-22 (r = 0.58; *p* < 0.002), but not IL-17A (Fig. 6e-h). Second, further analysis of the SF protein data in cohort 1 showed that SF IL-23 levels did strongly correlate with IL-17F (r = 0.643; *p* < 0.0001), IL-22 (r = 0.606; *p* < 0.0001), and IL-21 (r = 0.698; *p* < 0.0001) but not with IL-17A (*p* = 0.58) (Fig. 7). Confirming the specificity of these findings, IL-23 SF levels were not correlated to the Th1 cytokines IL-2 and IFNγ (data not shown).

In an attempt to explore this striking discrepancy between IL-17A and other Th17 cytokines, we determined the expression of key T cell transcription factors by qPCR in the third cohort of synovial tissue samples. Despite the small number of samples in this analysis, there was a clear correlation between CD21L expression and expression of GATA3 (r = 0.80; *p* = 0.001), TBX21 (r = 0.62; *p* = 0.077), and FoxP3 (r = 0.75; *p* = 0.020), probably reflecting the higher T cell infiltration in ELN+ samples. Strikingly, however, there was no correlation between either CD21L expression or IL-23 expression and RORC.

## Discussion

Synovial ELN is a well-recognized microarchitectural feature of rheumatoid synovitis which clinical and biological significance remains, however, unclear. Based on indirect evidence, it has originally been suggested that ELN may play a role in the breach of peripheral tolerance and thereby may fast-track autoimmunity [25]. Subsequent studies in large cohorts, however, consistently reported that the presence and/or levels of autoantibodies such as RF and ACPA were not related to the synovial ELN [5, 13, 14]. Exploring other potential links between ELN and synovial inflammation, an original report indicated that the presence of lymphoid follicles was associated with high expression of IFNγ and IL-10 and low expression of IL-4 [26]. Gene expression profiling of synovial tissue with ELN could, however, not reproduce these data and, in contrast, found an upregulation of the IL-7 pathway, which was suggested to promote ELN [27]. The present study, which was designed to assess systematically the association between synovial ELN and cytokines potentially involved in the pathogenesis of RA, could not reproduce the association with IL-7 but identifies for the first time a robust association between synovial ELN and increased expression of IL-23 and downstream cytokines such as IL-17F, IL-21 and IL-22.

The validity of the findings is supported by several observations. First, the frequency of ELN, the absence of relationship with clinical features such as disease duration and serological features such as RF and ACPA, and the association with higher levels of lymphocytic infiltration found in this study are perfectly in line with previously published data [5, 13, 14]. Second, the correlation between synovial ELN and activation of the IL-23 axis was technically and biologically reproducible using protein and mRNA approaches, using histological and molecular characterization of ELN, and across independent sample sets. The latter exclude false-positive results based on multiple comparisons or single outliers in the first cohort. Moreover, correlation analysis in the first cohort and confirmation in the second cohort (where DAS28 and CRP were similar in ELN+ and ELN- patients) confirmed that the association between synovial ELN and IL-23 was not biased by disease activity. Third, the robust correlation between ELN and IL-23 was recently also found by us in psoriatic arthritis (PsA) [28]. Finally, this association was specific as there was no significant correlation between ELN and other proinflammatory cytokines involved in RA synovial inflammation such as IL-6 and TNF.

The potential meaning and function of the described association between synovial ELN and activation of the IL-23 should be interpreted cautiously. Our immunopathological approach of synovitis allows the detection of meaningful disease features but its descriptive nature precludes causal interpretation without additional functional experiments in vitro and in animal models to investigate whether ELN promotes activation of the IL-23 axis or, alternatively, IL-23/IL-17 cytokines drive ELN and germinal center reactions. Interestingly, a series of elegant studies in experimental arthritis demonstrated that IL-17-producing Th cells and IL-17 orchestrate autoreactive germinal center development in autoimmune BXD2 mice [29, 30]. This was confirmed by studies in experimental autoimmune encephalomyelitis indicating that Th17 cells are effective B cell helpers and induce ELN in the inflamed central nervous system [31, 32]. Additionally, a recent report indicates that Th17 cells can acquire a follicular helper T cell phenotype and induced immunoglobulin A (IgA)-producing germinal center B cells in the intestinal Peyer’s patches [33]. These experimental data emphasize the functional role of the IL-23/IL-17 axis in germinal center reactions in tertiary and ectopic lymphoid structures. In the setting of ELN in human RA synovium, however, we did not observe preferential IL-23 p19 expression at the specific sites of ELN and failed to demonstrate an induction of ELN-associated chemokines such as CCL21 and CXCL13 by exposure of FLS to IL-23 and IL-17A.

Although we could thus not provide clear evidence that synovial ELN is driven by the IL-23 axis and the potential causal relationship between the two features thus remains unknown, our further analysis revealed two interesting features. First, immunofluorescence confirmed IL-23 p19 expression by synovial myeloid cells such as macrophages but also indicated p19 staining of vimentin-positive stromal cells. Previous studies reported that FLS can express p19 mRNA but not mRNA for the other IL-23 subunit, p40 [34, 35]. The significance of this finding remains to be further investigated, also in the context of ELN. Second, analysis of downstream cytokines of the IL-23 axis consistently indicated a correlation between ELN and protein and mRNA expression of IL-17F, IL-21, and IL-22 but, surprisingly, not IL-17A. It is increasingly recognized that IL-17A-producing cells, including canonical Th17 cells, are plastic and that the activation of the pathogenic IL-17A production module is transient and dependent on a variety of factors. One interpretation of our findings could thus be that the IL-23 axis is indeed activated in ELN+ synovitis but that this is not associated with activation of the pathogenic IL-17A production module in RA synovitis. The absence of correlation between ELN and RORC expression would be consistent with this interpretation. Alternatively, IL-23 could act on other cell types than canonical Th17 cells in the synovial tissue. Several immunopathological studies of human synovitis indeed reported a pronounced IL-17A staining of mast cells and, to a lesser degree, neutrophils while IL-17A-positive T cell were virtually undetectable [36–39]. There are no reports on the cellular source of IL-17F or IL-22 in human synovium and a systemic analysis of synovial IL-23R-positive cells is still lacking due to the absence of reliable monoclonal tools. In the present study, we did not pursue further the identification of IL-17F-, IL-21- and IL-22-producing synovial cells as data of such immunohistochemical analyses remain difficult to interpret for several reasons. First, this approach indicates the presence of the protein but not its production as the cytokine may, for example, be accumulated on the surface of target cells expressing its specific receptor. Second, this technique may be inappropriate to detect cells that continuously produce and secrete a cytokine of interest as the amount of protein within these cells would fall below the detection limit. Finally, this approach may miss rare cell populations such as innate lymphoid cells which, despite their paucity, may have important pathophysiological effects. Taken together, the mandatory identification of the cellular source and potential co-expression of different cytokines such as IL-17A, IL-17F, IL-21 and IL-22 by single cells requires different technical approaches such as ex vivo FACS sorting of specific highly purified cell populations for mRNA and protein analysis for these cytokines. These ongoing experiments fall beyond the scope of the present study on ELN.

Although causal relationship between synovial ELN and activation of the IL-23/IL-17 cytokine axis remains to be demonstrated, our study reveals for the first time a robust biological correlate to the heterogeneity of the histological microarchitecture of synovitis. The potential relevance of this finding is twofold. First, ELN has not only been observed in inflamed synovium but in many other chronically inflamed tissues affected by autoimmune diseases as well as chronic infections. The fact that we found the association with IL-23 in both RA and PsA [28] raise the hypothesis that this may be a global feature of ELN, which is not restricted to a particular disease or tissue. Second, phase two trials with monoclonal antibodies targeting IL-17A showed only modest signs of efficacy in RA [40–42]. Moreover, the response was not universal but heterogeneous between patients. Our observations raise the question whether the presence of synovial ELN, which is strongly associated with cytokines of the IL-23/IL-17 axis, could reflect this therapeutic heterogeneity and whether targeting other cytokines of the IL-23 axis, including IL-17F and IL-22, may provide additional clinical benefit. Prospective studies with baseline histological evaluation of ELN and comparing treatment with drugs targeting different cytokines of the IL-23 axis are warranted to address this hypothesis.

## Conclusions

The present study, aimed to assess the association between synovial ELN and cytokines potentially involved in the pathogenesis of RA, identifies for the first time a robust association between synovial ELN and increased expression of IL-23 and downstream cytokines such as IL-17F, IL-21 and IL-22. These findings could reflect the heterogeneity observed in the response to monoclonal antibodies targeting IL17A in phase II clinical trials in RA, and raise the question whether targeting other cytokines of the IL-23 axis, including IL-17F and IL-22, may provide additional clinical benefit.

## Additional file

Additional file 1: Figure S1. Immunohistological staining of IL-23p19 (1 ug) and istoype control (IC) in synovial membrane of RA patients. Magnification × 20.

## Abbreviations

ACPA: anti-citrullinated peptide antibodies
ACR: American College of Rheumatology
Ahr: aryl hydrocarbon receptor
Bcl6: B-cell CLL/lymphoma 6
CCL: CC chemokine ligand
CD21L: CD21 long isoform
cDNA: complementary deoxyribonucleic acid
CRP: C-reactive protein
CXCL: CXC chemokine ligand
DAPI: 4′,6-diamidino-2-phenylindole
DAS28: 28-joint disease activity score
DMARDs: disease-modifying antirheumatic drugs
EDTA: ethylenediaminetetraacetic acid
ELISA: enzyme-linked immunosorbent assay
ELN: ectopic lymphoid neogenesis
ESR: erythrocyte sedimentation rate
FBS: fetal bovine serum
FDC: follicular dendritic cells
FLS: fibroblast-like synoviocytes
FoxP3: forkhead box P3
GAPDH: human glyceraldehyde-3-phosphate dehydrogenase
Gata3: GATA binding protein 3
GM-CSF: granulocyte macrophage colony-stimulating factor
HEV: high endothelial venules
IFNγ: interferon gamma
IgA: immunoglobulin A
IL: interleukin
IQR: interquartile range
LTβ: lymphotoxin beta
mRNA: messenger ribonucleic acid
PCR: polymerase chain reaction
PNAd: peripheral lymph node addressin
PsA: psoriatic arthritis
qRT-PCR: quantitative real-time PCR
RA: rheumatoid arthritis
RF: rheumatoid factor
RorC: RAR-related orphan receptor C
SF: synovial fluid
SJC: swelling joint count
ST: synovial tissue
TBX21: T-box 21
TGF-β1: transforming growth factor beta 1
Th: T helper
TJC: tender joint count
TNF: tumor necrosis factor
