# Supplementary figures and images for: Ectopic lymphoid neogenesis is strongly associated with activation of the IL-23 pathway in rheumatoid synovitis
Source: Arthritis Res Ther. 2015 Jul 9;17(1):173. doi: 10.1186/s13075-015-0688-0 (PMC4496927; doi:10.1186/s13075-015-0688-0)

## Slide 1
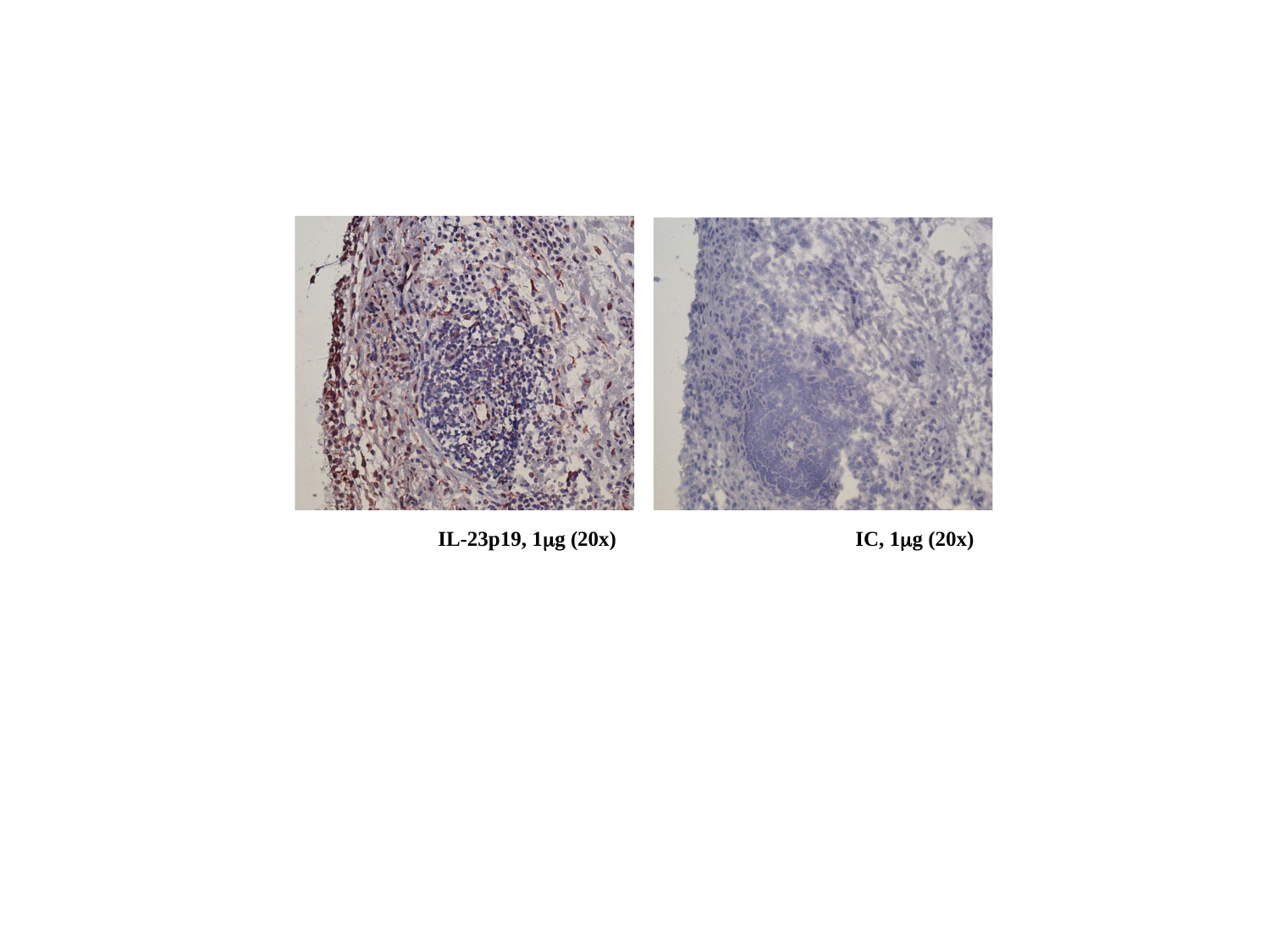

IL-23p19, 1mg (20x)
IC, 1mg (20x)

Supplement: Additional file 1: Figure S1. — Immunohistological staining of IL-23p19 (1 ug) and istoype control (IC) in synovial membrane of RA patients. Magnification × 20. [file 13075_2015_688_MOESM1_ESM.pptx]
